# Use of Bayesian Inference in Crystallographic Structure Refinement via Full Diffraction Profile Analysis

**DOI:** 10.1038/srep31625

**Published:** 2016-08-23

**Authors:** Chris M. Fancher, Zhen Han, Igor Levin, Katharine Page, Brian J. Reich, Ralph C. Smith, Alyson G. Wilson, Jacob L. Jones

**Affiliations:** 1 Department of Materials Science and Engineering, North Carolina State University, Raleigh, North Carolina 27695, USA; 2 Department of Statistics, North Carolina State University, Raleigh, North Carolina 27695, USA; 3 Materials Measurement Science Division, National Institute of Standards and Technology, Gaithersburg, Maryland 20899, USA; 4 Neutron Scattering Science Directorate, Oak Ridge National Laboratory, Oak Ridge, Tennessee 37831, USA; 5 Department of Mathematics, North Carolina State University, Raleigh, North Carolina 27695, USA

## Abstract

A Bayesian inference method for refining crystallographic structures is presented. The distribution of model parameters is stochastically sampled using Markov chain Monte Carlo. Posterior probability distributions are constructed for all model parameters to properly quantify uncertainty by appropriately modeling the heteroskedasticity and correlation of the error structure. The proposed method is demonstrated by analyzing a National Institute of Standards and Technology silicon standard reference material. The results obtained by Bayesian inference are compared with those determined by Rietveld refinement. Posterior probability distributions of model parameters provide both estimates and uncertainties. The new method better estimates the true uncertainties in the model as compared to the Rietveld method.

X-ray diffraction is a powerful technique for characterizing the atomic structure of materials. Diffraction relies on scattering of X-rays from planes of atoms resulting in either constructive or destructive interference. Figure 1 shows example peaks observed in diffraction patterns, and the inset is a schematic of X-ray scattering from planes of atoms that results in constructive interferences. The constructive interference is observed in the diffraction pattern as intensity vs scattering angle (2*θ*). Over the past 100 years a variety of approaches have been developed to determine and refine the crystallographic structure from single crystal and powder diffraction patterns^1^,^2^. The pervasive application of these approaches is evidenced by over 200,000 structures being cataloged in the International Centre for Diffraction Data Database. Moving forward, X-ray techniques and analysis methods will continue to advance, particularly in the areas of combining data from different experimental or theoretical sources^3^, and *in situ* measurements during materials’ processing or performance^1^,^2^,^4^. It is also expected that new crystallographic structures will be used in global materials databases for property prediction, as articulated by the Materials Genome Initiative (MGI)^5^,^6^.

In diffraction, observed intensities (*Y*_*i*_) at angle 2*θ*_*i*_ can be represented as intensities from the material of interest (*f*(2*θ*_*i*_ | *α*)), background scattering (*b*(2*θ*_*i*_ | *γ*)), and errors (*ε*_*i*_), where *i* represents the *i*^*th*^ data point in the measurement range. We write the observed intensity as


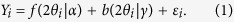


Here *α* is a collection of structural, microstructural, and instrumental parameters that determines the scattered intensity from a sample, and *γ* are parameters used to construct the background scattering. Most current approaches in crystallographic refinement infer the structure by minimizing the difference between a calculated and experimental pattern. The difference is minimized by adjusting model parameters. One example is the Rietveld method^7^,^8^, in which the structure is refined in a least-squares (LSQ) minimization routine using a weighted sum of squares residual:


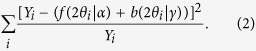


This minimization procedure results in estimates for each model parameter *α* and background parameter *γ*, and large-sample theory can be used to construct standard errors for these estimates.

While immensely powerful and widely applied, the Rietveld method can be subject to errors associated with false minima and uncertainty quantification^8^,^9^. In recent years, stochastic global optimization methods have been developed to improve the likelihood of reaching the global minimum^10^,^11^,^12^,^13^,^14^,^15^,^16^. However, the Rietveld method, and more recently developed stochastic methods, do not allow for the extraction of probability distributions of model parameters without prior knowledge of the distribution, a limitation that precludes the characterization of parameters in which a distribution is expected, e.g., unit cell descriptors (a, b, c, *α*, *β*, and *γ*). It is also well known that the Rietveld method handles statistical uncertainties using Hessian propagation^9^, and accounts for systematic errors by multiplying the resulting standard uncertainty in model parameters by the goodness of fit (i.e. scaling)^17^,^18^. The practice of scaling the obtained standard uncertainty is an approximation for the effect of systematic errors on uncertainty in model parameters^18^. Instead, a more complete model that formally accounts for systematic errors may obtain standard uncertainties without scaling.

The application of alternative statistical approaches can address these deficiencies. For example, Bayesian statistical inference^19^ can be used to compute posterior probability distributions of model parameters. Though not yet widely applied in full profile refinement for atomic structure determination, Bayesian statistical inference has been used in related areas of crystallography and measurement science^17^,^20^,^21^,^22^,^23^,^24^,^25^,^26^,^27^,^28^. For example, Wiessner and Angerer demonstrated the use of Bayesian approaches to determine probability distributions in the phase fractions of austenite and martensite during heating and cooling of a steel sample^29^. Recently, Gagin and Levin developed a Bayesian approach to account for unknown systematic errors in Rietveld refinements. Their method, paired with a least-squares minimization algorithm provided significantly more accurate estimates of structural parameters and their uncertainties than the standard analysis^17^.

In this paper, we introduce and employ an alternative and unique Bayesian statistical approach to solve the crystallographic structure refinement problem. Similar to the Rietveld method, a diffraction pattern is calculated from a modeled crystallographic unit cell. However the difference between the modeled and measured diffraction pattern is not minimized to reach single point value estimates of model parameters. Instead, the parameter space is explored by sampling multiple combinations of model parameters using a Markov chain Monte Carlo (MCMC) algorithm^30^. The results of the analysis are the posterior probability distributions of all modeled parameters, which yield both estimates of all parameters and quantifiable uncertainty. This work introduces a formal framework to account for unknown systematic errors (e.g. those manifested by correlated residuals), correlated and complex error variance patterns to help solve the problem of uncertainty quantification^9^, and a Monte Carlo simulation study that verifies the statistical validity of this approach. Measures of uncertainty are important for formally testing for differences between samples, comparing properties of the new sample to a historical value, and determining whether more data is required to obtain reliable results. While introduced in the present work using X-ray diffraction of a National Institute of Standards and Technology (NIST) standard reference material (SRM), the present approach can be readily adopted for use with data from other measurement probes and materials and should serve as a new structure refinement approach.

## Results

### Data collection and Rietveld analysis

A high resolution synchrotron diffraction pattern of a NIST Silicon standard (SRM 640d) was measured at the state-of-the art beamline 11-BM-B of the Advanced Photon Source (APS) at Argonne National Laboratory. The sample was held at constant temperature (22.5 °C) during the measurement. A Rietveld refinement analysis was first performed as a reference for a Bayesian approach. The execution of a Rietveld refinement also provides starting values for the MCMC algorithm.

The software package General Structure Analysis Software-II (GSAS-II) (version 0.2.0 revision 1466)^31^ was used to complete the Rietveld analysis. Traditionally, estimates are obtained by weighted least-squares minimization with weights proportional to the inverse of the intensity (Equation 1). The lattice parameter was fixed to the NIST reported value parameter (5.43123(8) Å at 22.5 °C)^32^,^33^. The Finger-Cox-Jephcoat peak profile function was used to account for axial divergence. Reported instrumental parameters (Caglioti (U = 1.163, V = −0.126, and W = 0.063) and axial divergence (SH/L = 0.0011)) were used. The space group and Wycoff positions were set to that of diamond cubic, and the isotropic thermal parameter (*U*_*iso*_) was initially set to be 0.001 (Å^2^). The parameter refinement sequence suggested by Young was followed to facilitate optimization and reduce the risk for nonconvergence of the least squares routine^8^. Additionally, Rietveld analyses were completed using various background orders (4^*th*^, 5^*th*^, 10^*th*^, and 15^*th*^) to confirm that the use of high order polynomial background does not yield an unstable least-squares refinement. Parameters obtained from all refinements were within two standard uncertainties, demonstrating that the 15^*th*^ order Chebyshev polynomial used in this work does not inhibit the Rietveld refinement.

**Figure 2:**
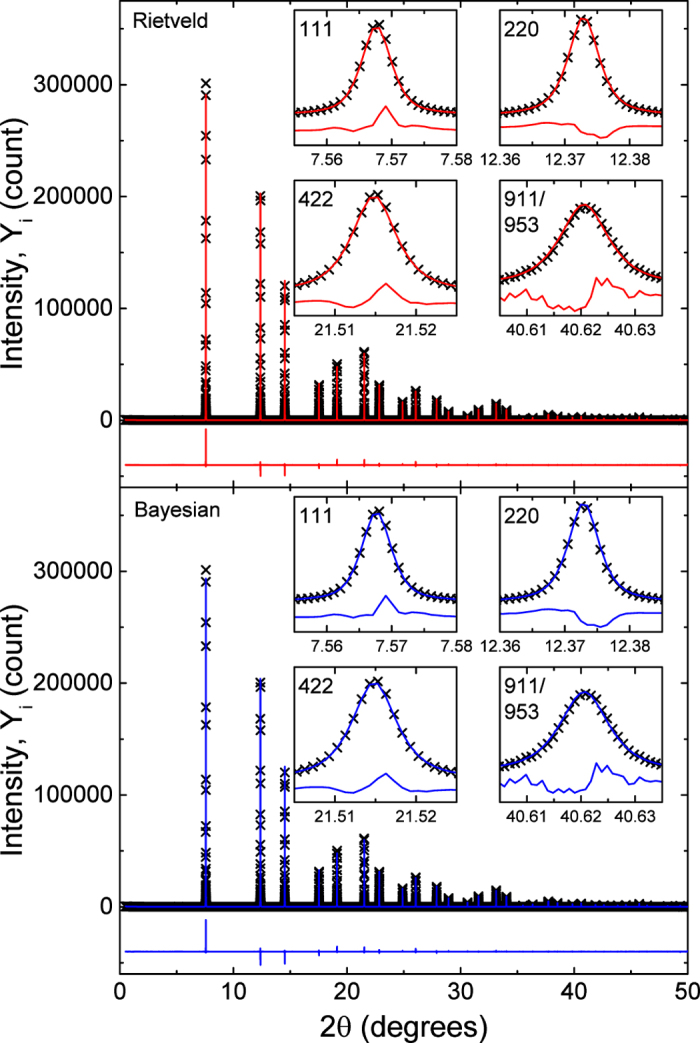
Representative Rietveld and Bayesian analysis results. Powder diffraction data of Si is compared with results of (top) a Rietveld analysis and (bottom) an average of the final 1000 MCMC samples. Insets of the 111, 220, 422, and 911/953 reflections show that the Bayesian inference and Rietveld approaches achieve a similar fit to the observed diffraction data.

A representative fit of the refinement result is presented in Fig. 2. The refined structural, microstructural, and instrumental parameters and goodness of fit indicators obtained from the Rietveld refinement are summarized in Table 1. A satisfactory fit could not be achieved without refining the Caglioti (U, V, and W) parameters. Instrumental parameters (Lx and Ly) associated with crystallite size and microstrain broadening were set to 0 and not refined; instead the crystallite size and microstrain broadening terms introduced in GSAS-II were used. The refined crystallite size (1.00006(6) *μ*m) and microstrain (0.0298(2)%) parameters were found to differ from the values reported in the NIST SRM 640D data sheet: 0.6 *μ*m and 0, respectively^32^,^33^. The origin of the discrepancy between the refined and NIST reported value might arise from a difference in resolution of the measured data (*i.e.* synchrotron vs laboratory) or the implementation of methods used to infer crystallite size and strain broadening contributions (*i.e.* GSAS-II vs TOPAS)^34^. It should be noted that the previous NIST Si standard (SRM 640c) did report a non-zero microstrain.

### Simulation study

Before describing a Bayesian analysis of the NIST silicon standard, we present a simulation study to illustrate the importance of properly modeling the variance and correlation of the residuals and compare the proposed method with more standard approaches. We performed this study on a face centered cubic structure as a model system. We have chosen to study a simple system with idealized data generation mechanisms in which we can model gross features of diffraction data to explore the impact of not accounting for correlated residuals when estimating parameters and uncertainties.

Our simulation study proceeds by generating synthetic data from a model with known parameter values. We then fit the synthetic data using Bayesian inference to obtain estimates and credible intervals for model parameters, and compare the estimated values and intervals with the true values used to generate the data. By repeating this process many times we can study the statistical properties of the method, including estimating the accuracy and coverage of credible intervals. In particular, we demonstrate that models that do not account for heteroskedastic and correlated residuals give poor estimates of model parameters, and that the actual coverage of 90% credible intervals can be far less than 90%.

**Figure 3:**
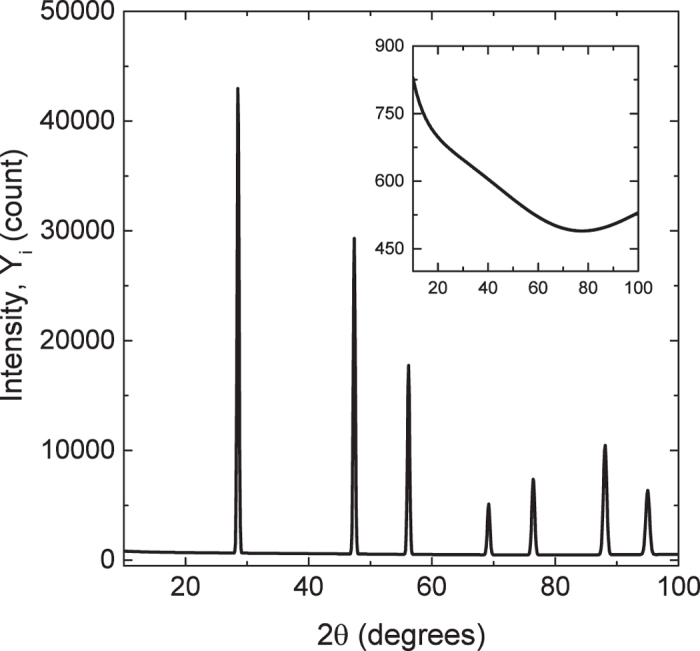
Synthetic data used for the simulation data. Simulated gold diffraction data and (inset) background used in the simulation study.

Synthetic data are generated from Equation 1. Observations are indexed by *i*, with *i* = 1,..., *n* = 901 observations made on a grid of 2*θ* spanning 10 to 100 degrees 2*θ* with 0.1 2*θ* step. An arbitrary background intensity (Fig. 3) is simulated to mimic a background that is typically observed in experiment


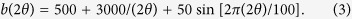


We set *f*(2*θ* | *α*) to be


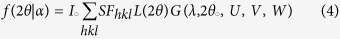


where *SF*_*hkl*_ and *L*(2*θ*) represent the structure factor for the *hkl* reflection and Lorentz-polarization correction, respectively. Structure factors of gold were utilized. The vector *α* is comprised of six parameters: incident X-ray intensity (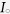); wavelength (*λ*); 2*θ* offset (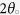); and Caglioti (U, V, and W) parameters. Diffraction peaks were modeled as Gaussian distributions with a full width half maximum that follows the Caglioti function^35^. Synthetic data were generated using 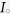 = 100, *λ* = 1.54 Å, 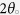 = 0.10, *U* = 0.10, *V* = −0.05, *W* = 0.03. The errors *ε*_*i*_ are normally distributed with variance *σ*^2^[*f*(2*θ*_*i*_ | *α*)/10000 + *γ*_0_], where *σ*^2^ = 2000^2^ and *γ*_0_ = 0.1. Errors on intensity are typically approximated using Poisson statistics, though in the limit of high counts this error can be approximated using a normal distribution. The stationary correlation between adjacent errors is set to *ρ* = 0.9, and autocorrelation between observations at angles 2*θ*_*i*_ and 2*θ*_*j*_ is *ρ*^|*i*−*j*|^. We generate *N* = 100 data sets from this model. Representative true and background intensities are shown in Fig. 3.

For each simulated data set, we fit four models that vary by their treatment of the residuals *ε*_*i*_. The goal of this simulation study is to examine the impact of fitting successively more complex models on the accuracy and precision of parameter estimates, as measured by relative mean squared error (RMSE) and the coverage of our credible intervals. We fit the model with independent residuals (correlation fixed at *ρ* = 0) and dependent residuals (*ρ* > 0). We also fit model with equal residual variance Var(*ε*_*i*_) = *σ*^2^ (analogous to least-squares minimization) and unequal residual variance Var(*ε*_*i*_) = *σ*^2^[*f*(2*θ*_*i*_ | *α*)/10000 + *γ*_0_] (analogous to weighted least-squares minimization). For all models, we capture the background process using a b-spline basis expansion with 20 degrees of freedom, which is sufficiently rich to capture the true background intensity. We use MCMC to generate 5,000 posterior samples, discarding the first 1,000 as burn-in. Additional details about the specification of the models, including prior distributions, can be found in the supplementary materials.

For each data set and each of the four models, we compute an estimate of the parameter (specifically, the posterior mean) and 90% (equal-tailed) credible interval for each parameter in *α*. A 90% credible interval should have a 90% probability of containing the true parameter. Let *α*_*j*_ be the true value of the *j*^*th*^ element of *α* and 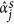 be posterior mean for data set *s* = 1,..., *N*. Methods are compared using relative mean squared error,


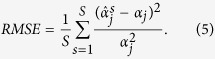


**Figure 4:**
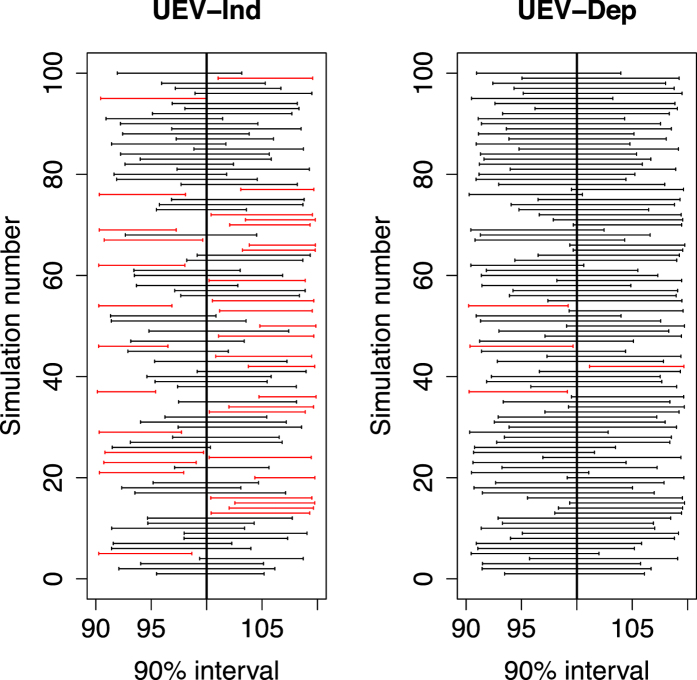
Coverage of the 90% interval. Illustration of the coverage in the simulation study. The plot shows the 90% posterior interval for 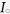 for all 100 simulated data sets and the unequal variance models with independent (left) and dependent (right) residuals. The intervals that exclude the true value (vertical line at 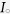 = 100) are show in red. The coverage percentage reported in Table 2 is the percent of the 100 intervals that include the true value.

We also compute the empirical coverage of the 90% intervals. That is, for each data set we compute the posterior 90% interval for each element of *α* and report the sample proportion of the *S* intervals that contain the true value *α*_*j*_ (as illustrated in Fig. 4). Methods with small RMSE for all parameters and coverage at or above 90% are preferred.

RMSE and coverage of the 90% credible intervals for each model are summarized in Table 2. The simplest model (independent residuals and constant (homoskedastic) variance) has the largest RMSE and lowest coverage of the 90% intervals. Including additional complexity (correlated residuals or variable (heteroskedastic) variance) decreases the RMSE and increases the coverage of the 90% interval. The coverage of the 90% intervals for the full model is near or above the nominal 90% level for all parameters while the coverage sinks far below 90% for simpler models. Therefore, we conclude that it is essential to adequately model the residual distribution of *ε* to obtain valid statistical inference. It is noted that the model implemented in Rietveld refinement method uses independent residuals and variable (heteroskedastic) variance. The Bayesian model we propose in the next section uses a more complex residual structure that should improve our estimates and credible intervals.

### Bayesian Analysis of the NIST silicon standard

**Figure 5:**
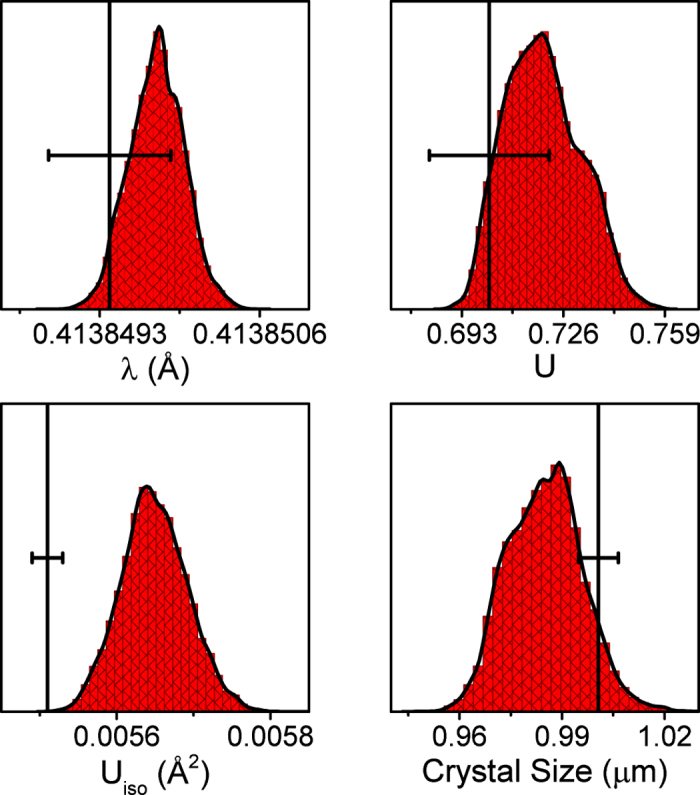
Comparison of posterior probability distribution and Rietveld estimates. Posterior probability distributions from Bayesian inference and corresponding point estimates (vertical lines) from Rietveld LSQ method with s.u.

Bayesian analyses begin by specifying a prior probability distribution that captures any knowledge about parameters that we have before the experiment. Based on observed data, this knowledge is updated to a posterior probability distribution, which reflects the resulting uncertainty about the model parameters. Figure 5 compares the posterior distributions for select model parameters (wavelength, U, *U*_*iso*_, and crystallite size) to point estimates and the corresponding standard uncertainty (s.u.) determined by Rietveld analysis. Posterior distributions for all model parameters are reported in supplemental information (Supplementary Figs 1, 2, 3, 4 and 5).

Figure 5 and Supplementary Figs 1, 2, 3, 4 and 5 demonstrate that model parameters estimated by Bayesian inference are in reasonable agreement with point estimates determined by Rietveld. With the exception of *U*_*iso*_ and scale, the posterior distributions overlap with the Rietveld point estimates. Figure 5 suggests that, for a NIST Si standard measured at 11-BM-B, the uncertainties in model parameters estimated by LSQ minimization are comparable to those estimated by Bayesian inference. In the case of the 2*θ* offset (zero) the s.u. is significantly larger than the uncertainty determined by Bayesian inference (Supplementary Fig. 5).

## Discussion

The two analysis approaches, Rietveld (non-Bayesian) and Bayesian inference, are fundamentally different. The most important distinction of the Bayesian approach is that prior distributions are created and propagated through the analysis to posterior distributions for parameters and predictive distributions for observables. A posterior distribution offers a richer set of information regarding the uncertainty in model parameters than the s.u. obtained from LSQ minimization. For example, a posterior distribution can be used to demonstrate that a model parameter has a multimodal or asymmetric distribution of values. This is evident in the posterior distribution of U (Fig. 5), *λ* (Fig. 5), crystal size (Fig. 5), V (Supplementary Fig. 3) and 2*θ* offset (Supplementary Fig. 5) where the posterior distributions are asymmetric.

Distributions of certain parameters can be determined from other refinement approaches. For example, the Whole Powder Pattern Modeling (WPPM) method has been used to model distributions of crystallite size using assumed distributional forms such as normal and lognormal^36^. However, the present approach enables the determination of general posterior densities without requiring these assumptions a priori.

To determine the quality of fit, crystallographers typically plot the modeled intensity against the observed intensities. Because Bayesian inference does not converge to a single-valued estimate for model parameters, any single diffraction profile generated from a MCMC sample should be thought of as one observation from an ensemble. One may construct a representative pattern from the ensemble by 1) choosing a single pattern or 2) averaging the calculated patterns from many MCMC samples. We use the second method and present in Fig. 2 a single pattern that is the average of the patterns produced using the parameters from the final 1000 MCMC samples. We employ method 2 because it incorporates correlations, asymmetries, and uncertainties in the parameter distributions when computing predicted patterns. Though this representation overly simplifies the result of the Bayesian approach, it does demonstrate that the modeled patterns match well to the measured data.

**Figure 6:**
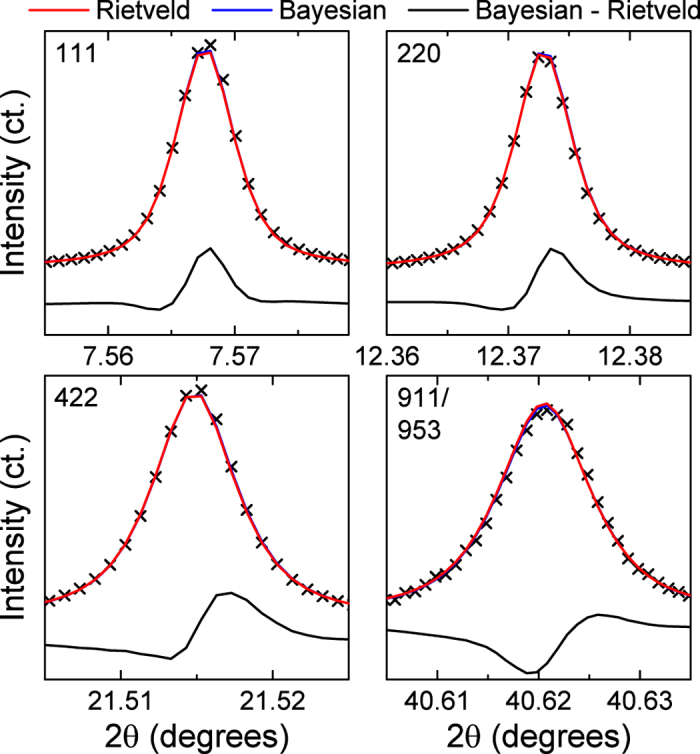
Direct comparison of graphical pattern fit from Bayesian and Rietveld analysis. Measured diffraction data are overlaid with Bayesian and Rietveld analysis results for the 111, 220, 422, and 911/953 reflections to highlight subtle differences in the fit quality. The difference (Bayesian - Rietveld) demonstrates that the Bayesian results better models the observed diffraction data (x).

Figure 2 shows the fit of the modeled pattern to the measured data for the Rietveld and Bayesian approaches. The insets in Fig. 2 highlight various reflections to illustrate that the Bayesian and Rietveld methods achieve a similar quality of fit. From Fig. 2 it is difficult to determine any subtle difference in the the fitting quality of the Bayesian and Rietveld analysis. The measured data and Rietveld and Bayesian results are overlaid, and a residual curve is also shown to highlight differences in the Rietveld and Bayesian results in Fig. 6. The difference curve demonstrates that Bayesian inference method better reproduces the observed diffraction profiles. This is evidenced by a positive and negative difference curve in regions where the Rietveld method under and over estimates the observed intensity. The 911/953 profile highlights that the Rietveld method underestimates the peak position, while Bayesian inference estimates a peak positions that more closely reproduces the observed peak position.

In addition to these graphical comparisons, Table 3 provides several numerical summaries of the Rietveld and Bayesian analyses. Each goodness-of-fit measure in Table 3 uses the discrepancy between the observed intensities *Y*_*i*_ and the calculated intensities 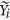. The goodness-of-fit measures differ by whether they use absolute or squared errors, and whether observations are weighted by the observed intensities. Results of the residual analysis presented in Table 3 demonstrate that the Bayesian inference method achieves a final result that more closely reproduces the measured diffraction data than the Rietveld method, as for each of the measurements of discrepancy, the results obtained by Bayesian inference are smaller. The improved performance provided by Bayesian inference is likely due to the manner in which parameters are optimized in Rietveld refinement. As described by Young^8^, Rietveld refinement employs a one-by-one turn-on sequence to facilitate optimization and avoid unstable least squares behavior, which can cause optimization to fail. In this sequence, uncorrelated parameters are optimized first, followed by those that are considered to be correlated or potentially less stable. Young notes that this strategy improves the robustness of the optimization procedure. The disadvantage is that coordinate descent in this manner is prone to termination in local minima, even when multiple initial values are employed. In contrast, the inherently stochastic nature of the MCMC approach mitigates being trapped in a false minimum because the parameter space (bounded by the priors) is randomly sampled and no turn-on sequence is employed. The Bayesian approach is thus less sensitive to the potential instabilities in parameters that can be observed in the Rietveld method. We note that the MCMC approach does not preclude chains from being trapped in local minima. However, the chance of becoming trapped in a local minimum can be reduced through the use of multiple initial starting values, careful consideration of algorithm diagnostics, and appropriate parameter transformation. We have employed all of these methods in the development of our algorithm, and details are discussed in the methods and supplementary materials.

The development and tuning of MCMC algorithms to perform Bayesian inference can be a complex process, especially when incorporating computations from other codes. In the example utilizing our approach, the computational expense of accurately modeling a diffraction pattern with 50,000 data points, 30 reflections, and complex peak shapes (required to account for axial divergence) is a substantial bottleneck for the MCMC analysis. At each MCMC iteration a model diffraction pattern must be calculated using GSAS-II for each of the ten model parameters. One thousand iterations required, on average, 900 s on an Intel CoreTM i5-3750. [The identification of any commercial product or trade name does not imply endorsement or recommendation by the National Institute of Standards and Technology.] To be conservative, we ran the MCMC algorithm for 100,000 iterations. Diagnostic plots, like those in Supplementary Figs 6, 7 and 8, show the convergence of the algorithm.

One particular advantage of Bayesian inference is its ability to incorporate prior knowledge. With the exception of rigid body constraints, there is no framework for incorporating prior knowledge about the sample of interest (e.g., crystallite size obtained by electron microscopy analysis) into a Rietveld analysis. A prior distribution, which makes a probabilistic specification of prior knowledge, is required in the Bayesian approach. While this can be defined by physical expectations at the outset of an analysis, it is also possible to employ iterative or hierarchical calculations in the Bayesian approach. For example, posterior distributions obtained from a Bayesian inference of LaB_6_ NIST SRM 660a can serve as priors for subsequent analyses. I.e., one might first complete a Bayesian analysis on a NIST SRM designed to quantify line broadening to obtain posterior distributions for instrumental parameters (e.g., wavelength, axial divergence, and Caglioti peak shape parameters). These results could be used in analyses of structurally more complex materials (such as relaxor ferroelectrics or high entropy alloys) where well-calibrated instrumental parameters are critical for obtaining posterior distributions that precisely describe the structure of the material of interest, for example, the lattice and atomic parameters (displacements, occupancies, and thermal factors)^8^. Additionally, the MCMC algorithm has an important advantage over a deterministic least-squares minimization: the ability to escape from local minima. The stochastic aspect of the MCMC algorithm enables the method to overcome barriers that would otherwise trap a least-squares method in a confined region of parameter space. This advantage makes the use of the present method advantageous despite its computationally-intensive nature.

In summary, we present a new Bayesian inference method for refining crystallographic structures. This approach is fundamentally different from that of the Rietveld method, which is currently the predominant analysis method for structure refinement. This analysis approach is applied to an example where the structure of a standard reference material is refined using synchrotron X-ray diffraction data. The approach can be readily adopted to refine other structures as well as integrating additional or different data sources (e.g., area detector patterns and neutron diffraction patterns). The Bayesian approach offers a richer description of the model and model uncertainties than has been available previously.

## Methods

### Data collection and Rietveld analysis

An X-ray wavelength of 0.413848 Å (30.1 keV) was utilized. Diffracted X-rays were measured using an array of twelve detectors with Si (111) analyzer crystals^37^,^38^. Measured data was merged into a single data set using previously reported methods^38^.

A 15^*th*^ order Chebyshev polynomial background was used to account for an irregular background due to amorphous scattering from the Kapton^®^ capillary. Parameters refined include the zero, profile shape (U, V, and W), scale, wavelength, isotropic displacement parameter (*U*_*iso*_), microstrain and crystallite size.

### Prior distributions for simulation study

Within the simulation study, the coefficients for the background model have prior distribution 
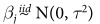
. The other prior distributions used are *σ*^2^, *τ*^2^ ~ InvGamma(0.01, 0.01), log(*γ*_0_) ~ N(0, 10^2^) (for the heteroskedastic models), *I*_*o*_ ~ Unif(90, 110), *λ* ~ Unif(1.5, 1.6), 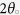 ~ Unif(0, 1), *U* ~ Unif(0.08, 0.12), *V* ~ Unif(−0.06, −0.03), *W* ~ Unif(0.015, 0.45), and *ρ* ~ Unif(0, 1) (for the models with correlated residuals).

### Bayesian inference

The Bayesian inference method uses Equation 1 to model the observed data. The background intensity is approximated using the spline basis expansion,


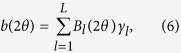


where *B*_*l*_ are known basis functions and *γ*_*l*_ are unknown coefficients that determine the shape of *b*. In our analysis we use b-spline basis functions. B-splines are a flexible piecewise polynomial functions used for curve fitting that provide results similar to higher degree polynomials while avoiding potential instabilities^39^. The coefficients *γ*_*l*_ have priors *γ*_*l*_ ~ Normal(0, *τ*^2^). This prior was selected because we do not have prior information about the background intensity; it does not imply that our resulting estimates are centered at 0. By treating these coefficients as part of the Bayesian hierarchical model we propagate uncertainty about the background correction through to the posterior distributions.

Since the counts are large, we approximate their distribution as Gaussian with variance depending on the mean. Specifically, the residuals are modeled as Gaussian with mean zero and variance Var(*ε*_*i*_) = *σ*^2^[*f*(2*θ*_*i*_ | *α*)/*C* + *γ*_0_], where *C* is a large constant to scale the variance. Therefore, the overall variance is determined by *σ*^2^, but the variance increases with the intensity *f*(2*θ*_*i*_ | *α*) ≥ 0. The constant *γ*_0_ > 0 is included so that the variance remains positive for observations with *f*(2*θ*_*i*_ | *α*) = 0. This approach is in contrast with the frequent assumption that the residual variance is proportional to the observed intensity, *Y*_*i*_, and is a common statistical approach for count data. In addition to heteroskedasticity, we account for dependence between the residuals for intensities with similar angles using an AR(1) process, so that correlation between residuals *h* observations is *ρ*^*h*^. The correlation parameter is given an uninformative prior and is estimated as part of the MCMC process. Autocorrelation in residuals could arise from background scattering. If the modeled background is lower-order than required to fit the diffuse scattering, then autocorrelation would be expected. Likewise, an imprecise peak position would lead to autocorrelation. This is because many data points are used to sample a smooth peak and the left and right hand sides of the peaks would both exhibit correlated residuals.

To complete the Bayesian model we specify prior distributions for the remaining parameters. As examples, the prior distribution for X-ray wavelength is uniform between 0.41 and 0.42, and the prior distribution for the *U*_*iso*_ is uniform between 0.0 and 0.1. The remaining parameters are summarized in Supplementary Table 1. For this analysis, we use *L* = 15 b-spline basis functions to mirror the Rietveld analysis.

Modeled diffraction patterns used for the Bayesian analysis were calculated using routines extracted from GSAS-II. A standalone Python class was developed to interface with GSAS-II routines utilized to calculate a diffraction pattern (i.e., peak intensity, position, and profile shape). This Python class enabled calculating a modeled diffraction pattern and modification of parameters that controls the modeled pattern (e.g., lattice parameters, atomic occupancy and position, *U*_*iso*_ factor, peak profile parameters, etc.). The Python code is included in a Supplementary note.

### MCMC Details

MCMC is an iterative, general-purpose algorithm to draw samples from a complex, multivariate distribution^30^: in this case, the posterior distribution of the parameters of the model. The algorithm requires starting values, which we initialize using the point estimates from the Rietveld refinement in our analyses. To improve convergence, we transform bounded parameters to take values on the real line and linearly transform correlated parameters. We run 100,000 iterations and verify convergence using standard diagnostics. Since the procedure is iterative, observations near the beginning of the chain (called *burn-in*) have not converged and are not draws from the appropriate distribution, so they are discarded. Here, the first 10,000 runs are discarded for burn-in. The posterior distributions are summarized using the posterior mean, which is calculated as the average of the posterior samples, and posterior credible intervals. Since a posterior distribution is a probability distribution, it integrates to 1. A p% credible interval is an interval over which the posterior distribution integrates to *p*/100.

Since MCMC is an iterative procedure, there are a variety of diagnostics used to diagnose when samples are being drawn from the desired distribution and how many samples should be discarded as “burn-in.” Supplementary Fig. 6 plots sample pairs for three parameters (and marginal density estimates). If the sample pairs indicate high correlation (e.g., the point clouds are elongated instead of round), this may indicate that the MCMC should be adjusted to allow for faster convergence. Supplementary Fig. 7 plots successive samples from a single parameter. These plots are termed *trace plots*. As convergence is reached, the samples stabilize around a value; the early samples are burn-in. This is also illustrated by Supplementary Fig. 8.

We perform MCMC sampling using an equivalent and convenient parameterization. The priors for the elements of *α* = (*α*_1_,...,*α*_*p*_) are independent and uniform *α*_*j*_ ~ Uniform(*l*_*j*_, *u*_*j*_), where the prior intervals (*l*_*j*_, *u*_*j*_) are chosen to give a scientifically plausible range of values. MCMC sampling is easier for parameters defined on the real line, so we perform computing using the parameterization *α*_*j*_ = *l*_*j*_ + (*u*_*j*_ − *l*_*j*_)Φ(*z*_*j*_), where Φ is the standard normal cumulative distribution function and *z*_*i*_ have standard normal priors. This induces a Uniform(*l*_*j*_, *u*_*j*_) prior for *α*_*j*_, and so the parameterization is equivalent to the desired model.

For *i* > 1 the auto-regression model can be written:


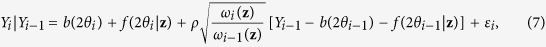


where **z** = (*z*_1_,..., *z*_*p*_), *ε*_*i*_ ~ Normal[0, *σ*^2^(1 − *ρ*^2^)*ω*_*i*_(**z**)] independent over *i*, and *ω*_*i*_(**z**) = *f*(2*θ*_*i*_ | **z**)/*C* + *γ*_0_. Since the first observation provides negligible information, we ignore the first observation and express the remaining *n* − 1 observations in matrix notation,


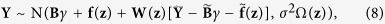


where **Y** = (*Y*_2_,..., *Y*_*n*_)^*T*^, **B** is the (*n* − 1) × *L* matrix of basis coefficients, 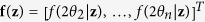, 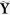, 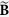, 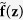 are lagged matrices, e.g., 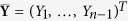, **W**(**z**) is diagonal with diagonal elements 
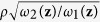
, …, 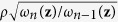, and Ω(**z**) is diagonal with diagonal elements (1 − *ρ*^2^)*ω*_2_(**z**), …, (1 − *ρ*^2^)*ω*_*n*_(**z**).

We explore the posterior by generating MCMC samples. MCMC sampling begins by specifying initial values for all parameters, *γ*, **Z**, *ρ*, *τ*^2^ and *σ*^2^. For each iteration of the algorithm, the parameters are updated in sequence conditioned on all other parameters. The parameters *γ*, *σ*^2^, *τ*^2^ have conjugate full conditional distributions and are thus updated using Gibbs sampling. The full conditional distributions are


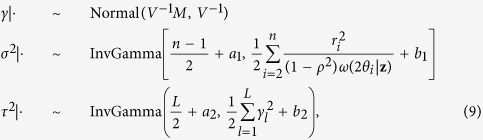


where 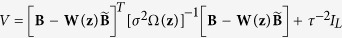, 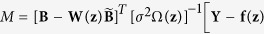

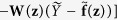
, 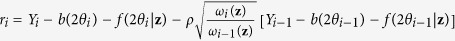 and the priors are *σ*^2^ ~ InvGamma(*a*_1_, *b*_1_) and *τ*^2^ ~ InvGamma(*a*_2_, *b*_2_).

The remaining parameters *ρ* and **z** do not have conjugate full conditionals and are thus updated using Metropolis sampling. We use random-walk Gaussian candidate distributions for both *ρ* and **z**. The standard deviation for *ρ*’s candidate distribution is tuned to give acceptance probability near 0.4; candidates outside the prior range (0, 1) are discarded. The parameter vector **z** is updated as a block. The proposal covariance matrix is *c* Δ, where *c* is a tuning parameter selected to give acceptance probability near 0.4 and the matrix Δ is taken to be the posterior covariance matrix of **z** from an initial MCMC sample.

## Additional Information

**How to cite this article**: Fancher, C. M. *et al*. Use of Bayesian Inference in Crystallographic Structure Refinement via Full Diffraction Profile Analysis. *Sci. Rep.* **6**, 31625; doi: 10.1038/srep31625 (2016).

## Supplementary Material

Supplementary Information

## Figures and Tables

**Figure 1:**
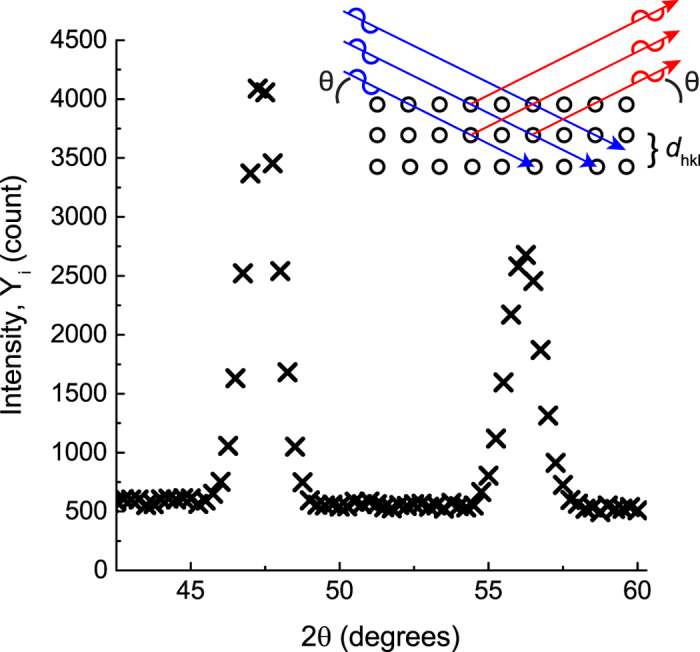
Example peaks observed in an X-ray diffraction pattern and schematic of X-ray scattering from atoms. Constructive interference of X-rays scattered from planes of atoms results in observed peaks at various scattering angle (2*θ*), which is characteristic of the interplanar spacing. The inset is a schematic illustration of X-rays incident at an angle *θ* that results in constructive interference from a periodic array of atoms at an angle *θ* from the plane of atoms.

**Table 1:** Summary of refined structural parameters, atomic positions and occupancies, and goodness of fit for the Rietveld refinement.

a (Å)	Crystal size (*μ*m)	microstrain (%*100)	*λ* (Å)	Profile fit
				*R*_*p*_ = 5.85%
5.43123	1.0006(6)	2.98(2)	0.4138490(5)	*R*_*w*_ = 8.28%
				*χ*^2^ = 2.02
	Site positions			
	x	y	z	*U*_*iso*_(Å^2^)
Si	0.125	0.125	0.125	0.00551(2)
Peak shape parameters				
U	V		W	
0.702(19)	−0.242(6)		0.0322(5)	

**Table 2:** Simulation study results for models with residuals that are independent (“Ind”) and dependent (“Dep”), and with equal-variance (“EV”) and unequal variance (“UEV”).

Parameter	Ind-EV	Ind-UEV	Dep-EV	Dep-UEV
(a) Relative mean squared error				
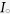	0.32 (0.03)	0.20 (0.02)	0.38 (0.03)	0.12 (0.01)
100*λ*	0.30 (0.14)	0.16 (0.02)	27.08 (9.80)	0.14 (0.02)
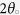	7.81 (3.53)	4.47 (0.68)	514.02 (174.25)	3.94 (0.70)
*U*	1.05 (0.10)	0.63 (0.06)	0.83 (0.08)	0.23 (0.03)
*V*	3.41 (0.37)	1.25 (0.17)	3.27 (0.38)	0.78 (0.08)
*W*	2.06 (0.28)	1.06 (0.13)	3.47 (0.61)	0.58 (0.08)
(b) Coverage of 90% intervals				
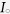	17 (4)	64 (5)	23 (4)	96 (2)
100*λ*	32 (5)	65 (5)	30 (5)	87 (3)
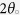	31 (5)	69 (5)	25 (4)	85 (4)
*U*	61 (5)	86 (3)	70 (5)	100 (0)
*V*	55 (5)	98 (1)	69 (5)	100 (0)
*W*	41 (5)	79 (4)	48 (5)	95 (2)

Results are reported as the mean (standard error) over the *S* simulated data sets; all values are multiplied by 100, and results are reported for 100*λ* rather than *λ*.

**Table 3:** Summary of residual analysis using results from both the Rietveld and Bayesian approaches.

Residual Method	Bayes/ Rietveld
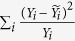	0.79
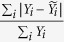	0.90
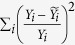	0.89
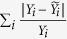	0.85
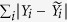	0.89
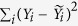	0.92
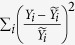	0.64
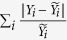	0.80
