# Supplementary material for: Use of Bayesian Inference in Crystallographic Structure Refinement via Full Diffraction Profile Analysis
Source: Sci Rep. 2016 Aug 23;6:31625. doi: 10.1038/srep31625 (PMC4994022; doi:10.1038/srep31625)
Supplement: Supplementary Information [file srep31625-s1.pdf]

# Use of Bayesian Inference in Crystallographic Structure Refinement via Full Diffraction Profile Analysis

**Chris M. Fancher[1], Zhen Han[2], Igor Levin[3], Katharine Page[4], Brian J. Reich[2], Ralph C. Smith[5], Alyson G. Wilson[2], and Jacob L. Jones[1,*]**

[1]Department of Materials Science and Engineering, North Carolina State University, Raleigh, North Carolina 27695, USA
[2]Department of Statistics, North Carolina State University, Raleigh, North Carolina 27695, USA
[3]Materials Measurement Science Division, National Institute of Standards and Technology, Gaithersburg, Maryland 20899, USA
[4]Neutron Scattering Science Directorate, Oak Ridge National Laboratory, Oak Ridge, Tennessee 37831, USA
[5]Department of Mathematics, North Carolina State University, Raleigh, North Carolina 27695, USA
[*]jacob_jones@ncsu.edu

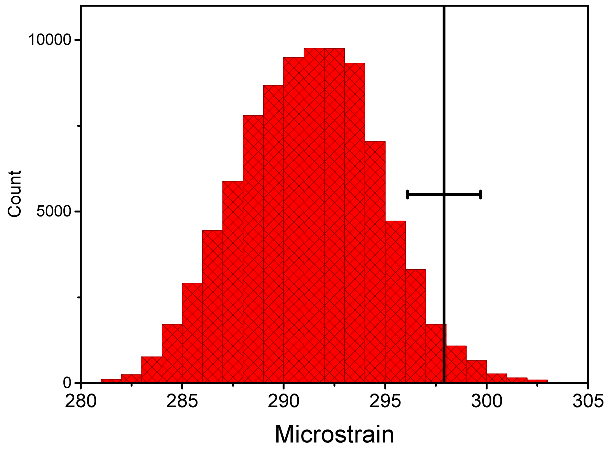


**Supplementary Figure 1.** Posterior probably distributions from Bayesian inference and corresponding point estimates (vertical lines) from Rietveld LSQ method with estimated errors.

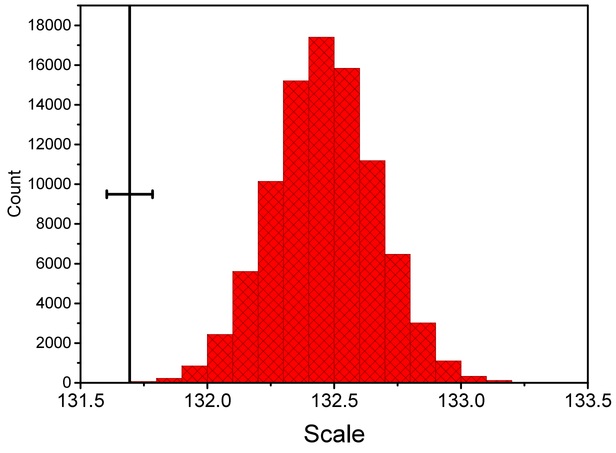


**Supplementary Figure 2.** Posterior probably distributions from Bayesian inference and corresponding point estimates (vertical lines) from Rietveld LSQ method with estimated errors.

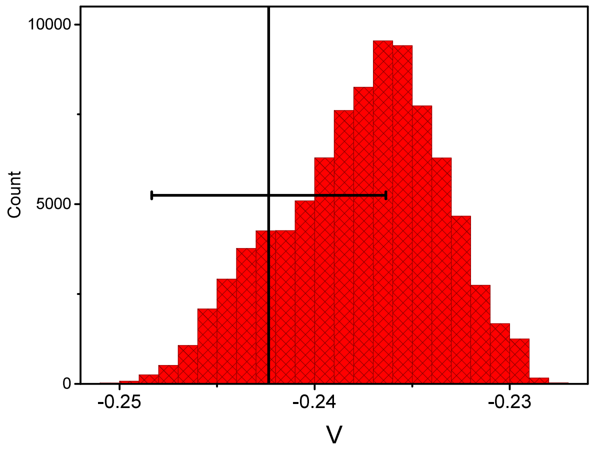


**Supplementary Figure 3.** Posterior probably distributions from Bayesian inference and corresponding point estimates (vertical lines) from Rietveld LSQ method with estimated errors.

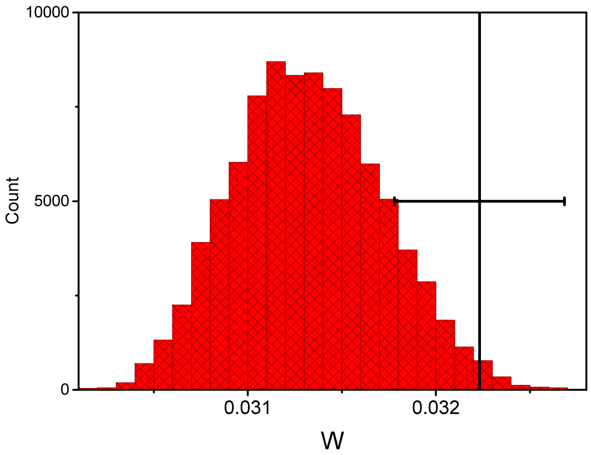


**Supplementary Figure 4.** Posterior probably distributions from Bayesian inference and corresponding point estimates (vertical lines) from Rietveld LSQ method with estimated errors.

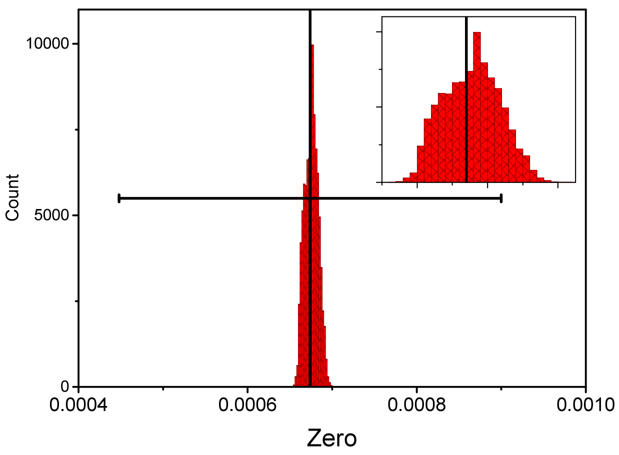


**Supplementary Figure 5.** Posterior probably distributions from Bayesian inference and corresponding point estimates (vertical lines) from Rietveld LSQ method with estimated errors.

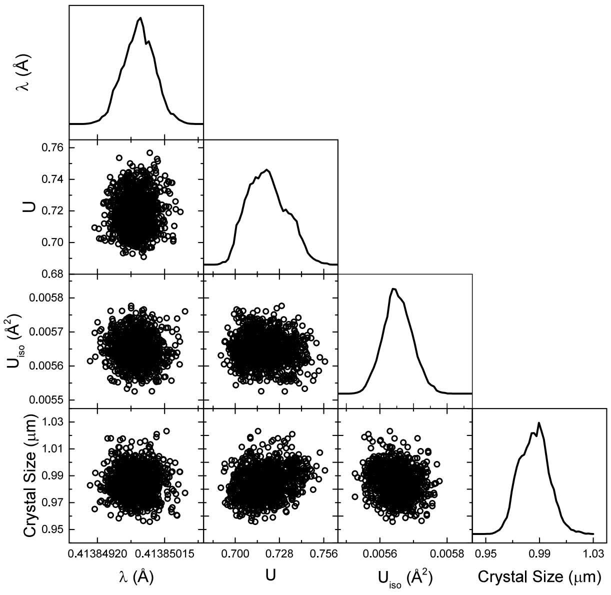


**Supplementary Figure 6.** Pairs plots of model parameters are a common diagnostic used to identify correlations among parameters that might lead to slow mixing and convergence of the MCMC algorithm.

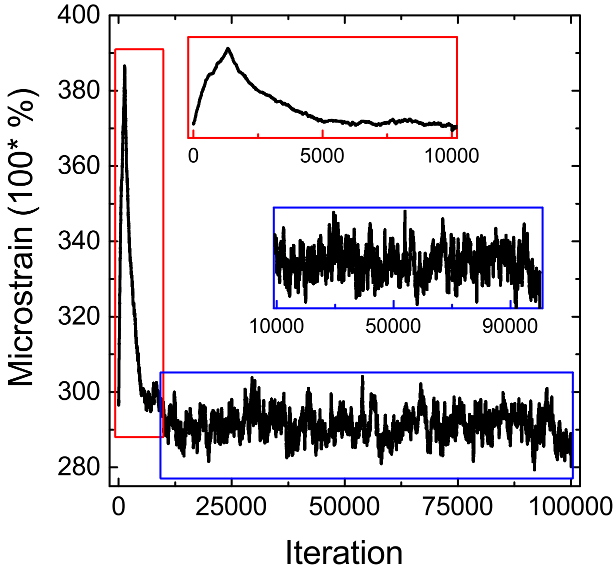


**Supplementary Figure 7.** Traceplot of the MCMC samples for microstrain illustrates the behavior of the MCMC algorithm as parameter space is stochastically sampled. Initially the sampled values vary widely until the analysis reaches a stable region of parameter space. These iterations are referred to as burn-in and are discarded the subsequent analysis because these samples are not representative of the posterior distribution. The insets are shown to highlight the behavior of the MCMC sample before (red) and after (blue) burn-in.

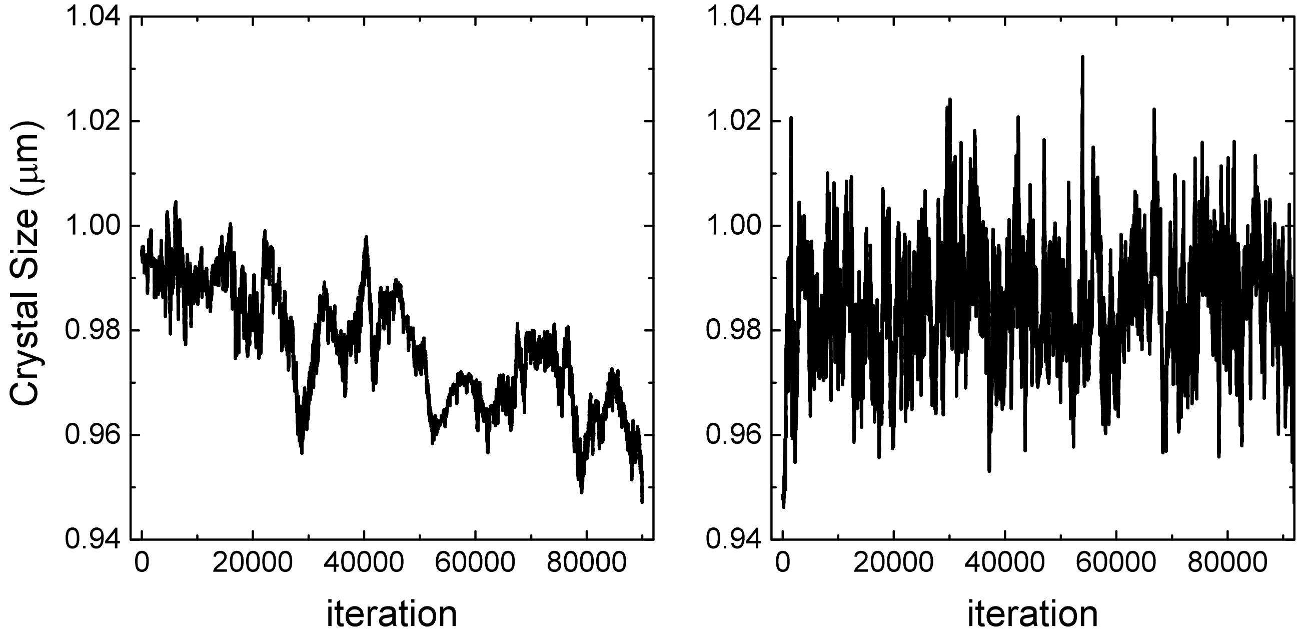


**Supplementary Figure 8.** Example trace plots of MCMC samples that (left) have not reach convergence and (right) have converged. The trend that the MCMC samples decreases with iteration number indicates that the samples have not reached a stable point. The plot on the left is from a preliminary run of the MCMC and is not used in any reported calculations.

**Supplementary Table 1.** Summary of prior distributions and constants used in the MCMC analysis.

| Parameter | Prior Distribution/Fixed Value |
|---|---|
| Wavelength | Uniform(0.41, 0.42) |
| U | Uniform(0.1, 1.5) |
| V | Uniform(-0.35, -0.04) |
| W | Uniform(0.0, 0.1) |
| Scale | Uniform(0, 500) |
| Zero | Uniform(-0.1, 0.1) |
| Microstrain | Uniform(0, 600) |
| Crystallite Size | Uniform(0, 2) |
| $U_{iso}$ | Uniform(0.0, 0.1) |
| B-spline parameters ($\gamma$) | Normal$(0, \tau^2)$ |
| $\tau^2$ | InverseGamma(0.1,0.1) |
| $\sigma^2$ | InverseGamma(0.1,0.1) |
| $\gamma_0$ | LogNormal(0,1) |
| Polarization | 0.99 |
| Absorption | 0.1 |
| X | 0 |
| Y | 0 |
| d | 5.431230 |
| C | 10,000 |

```
#Here load up the package outside of the function to make it a one time thing
#minimal cost because this is only loaded once
#import time
import GSASIIstrIO as G2stIO
import GSASIIIO as G2IO
import GSASIIstrMath as G2stMth
import GSASIIpath
import pylab as plt

class Calculator:

    '''
        ################################################## How it works ####################
        # Variables are stored in various python dictionaries
        # (parmDict, calcControls, Phases, and Histograms)
        #
        # Variables of interest that are stored in parmDict include:
        # Background parameters: :0:Back:x (x represent the order)
        # X-ray absorption coef: :0:Absorption
        # X-ray wavelength: :0:Lam
        # Intensity scale factor: :0:Scale
        # 2-theta offset: :0:Zero
        # Various peak parameters: :0:U, :0:V, :0:W, :0:L, :0:X, :0:SH/L
        #
        # Variables of interest that are stored in calcControls include:
        # Model for background: :0:bakType (default is chebyschev)
        # available models are:
        # chebyschev (default), cosine, lin interpolate, inv interpolate, and log interpolate
        #
        # Phases contains information about the phases in the material
        # (Space group, atom positions, Debye-Waller factors, preferred orientation, ....)
        # Change between isotropic and anisotropic Debye-Waller factors:
        # Phases[Phases.keys()[0]]['Atoms'][X][9] = 'I' (isotropic) or 'A' (anisotropic)
        # [X represents atom number (0 to n-1)]
        # Debye-Waller factors are controled by:
        # Phases[Phases.keys()[0]]['Atoms'][X][10-16] (isotropic case only used element 10)
        #
        # Histograms contains information about the diffraction data file (DO NOT CHANGE)
        #

    '''
    def __init__(self,GPXfile=None):

        '''
            Initialize the setup using an existing GPXfile
        '''

        if not GPXfile:
            #TO DO: Raise name error
            raise NameError('Must␣input␣some␣GPX␣file')

        # Initallizing variables from input file GPXfile
        #Code from Chris
        #time0 = time.time()
        varyList = []
```

```
        parmDict = {}
        Controls = G2stIO.GetControls(GPXfile)
        calcControls = {}
        calcControls.update(Controls)
        constrDict,fixedList = G2stIO.GetConstraints(GPXfile)
        restraintDict = G2stIO.GetRestraints(GPXfile)
        Histograms,Phases = G2stIO.GetUsedHistogramsAndPhases(GPXfile)
        rigidbodyDict = G2stIO.GetRigidBodies(GPXfile)
        rbIds = rigidbodyDict.get('RBIds',{'Vector':[],'Residue':[]})
        rbVary,rbDict = G2stIO.GetRigidBodyModels(rigidbodyDict)

        if GSASIIpath.GetVersionNumber() > 1500:
           Natoms,atomIndx,phaseVary,phaseDict,pawleyLookup,FFtables,BLtables, \
              maxSSwave = G2stIO.GetPhaseData(Phases,restraintDict,rbIds,Print=False)
        else:
           Natoms,atomIndx,phaseVary,phaseDict,pawleyLookup,FFtables,BLtables = \
                    G2stIO.GetPhaseData(Phases,restraintDict,rbIds,Print=False)

        calcControls['atomIndx'] = atomIndx
        calcControls['Natoms'] = Natoms
        calcControls['FFtables'] = FFtables
        calcControls['BLtables'] = BLtables
        hapVary,hapDict,controlDict = \
              G2stIO.GetHistogramPhaseData(Phases,Histograms,Print=False)
        calcControls.update(controlDict)
        histVary,histDict,controlDict = G2stIO.GetHistogramData(Histograms,Print=False)
        calcControls.update(controlDict)
        varyList = rbVary+phaseVary+hapVary+histVary
        parmDict.update(rbDict)
        parmDict.update(phaseDict)
        parmDict.update(hapDict)
        parmDict.update(histDict)
        G2stIO.GetFprime(calcControls,Histograms)
        varyListStart = tuple(varyList)
        # save the original varyList before dependent vars are removed

        #Save the instance parameters
        self._Histograms = Histograms
        self._varyList = varyList
        self._parmDict = parmDict
        self._Phases = Phases
        self._calcControls = calcControls
        self._pawleyLookup = pawleyLookup
        self._restraintDict = restraintDict
        self._rigidbodyDict = rigidbodyDict
        self._rbIds = rbIds
        self._tth = Histograms[Histograms.keys()[0]]['Data'][0][0:-1]


#print 'variable initallizing time: %.3fs...Successful'%(time.time()-time0)

    def Calculate(self):

        '''
           Calculate the f and g for the current parameter setup
```

```
    '''

    #time0 = time.time()
    '''
        #Load the parameters
        Histograms = self._Histograms
        varyList = self._varyList
        parmDict = self._parmDict
        Phases = self._Phases
        calcControls = self._calcControls
        pawleyLookup = self._pawleyLookup
        restraintDict = self._restraintDict
        rbIds = self._rbIds
    '''

    yc1,yb1 = G2stMth.getPowderProfile(self._parmDict,self._tth,self._varyList, \
                           self._Histograms[self._Histograms.keys()[0]], \
                           self._Phases,self._calcControls,self._pawleyLookup)

    # print 'Pattern calculation time: %.3fs'%(time.time()-time0)

    return [self._tth, yc1, yb1]

def CalculateDeriv(self):

    X = G2stMth.getPowderProfileDerv(self._parmDict,self._tth,self._varyList,\
                         self._Histograms[self._Histograms.keys()[0]],\
                         self._Phases,self._rigidbodyDict, \
                         self._calcControls,self._pawleyLookup)

    return X

def SaveData(self,OutputFileName):

    x, yc, yb = self.Calculate()

    outputFile = open( OutputFileName, 'w' )

    for i in xrange( len( x ) ):
        outputFile.write( str(x[i]) + '\t' + str(yc[i]) + '\t' + str(yb[i]) + '\n')

def Draw(self,SaveFigure=None):
    '''
        Calculate the f and g for the current parameter setup
    '''
    x, yc, yb = self.Calculate()

    plt.plot(x,yc+yb)

    if SaveFigure:
        plt.savefig(SaveFigure)
    else:
        plt.show()

def UpdateParameters(self, parmVarDict=None, controlVarDict=None, \
```

```
             phaseVarDict=None, histogramVarDict=None):
'''
   Update parameters in the current model
'''

if not(parmVarDict or controlVarDict or phaseVarDict):
   print 'No␣update␣specified...Abort'
   return 0

if histogramVarDict:
   for key1 in histogramVarDict.keys():
      for key2 in histogramVarDict[key1].keys():
         self._Histograms[self._Histograms.keys()[0]][key1][0][key2][1] = \
            histogramVarDict[key1][key2]

if phaseVarDict:
   for key1 in phaseVarDict:
      if key1 == 'Atoms':
         self._Phases[self._Phases.keys()[0]][key1][phaseVarDict[key1]\
            ['Atom']][phaseVarDict[key1]['i1']] = phaseVarDict[key1]['Val']
      else:
         for key2 in phaseVarDict[key1]:
            if key1 == 'Histograms':
               self._Phases[self._Phases.keys()[0]][key1]\
                  [self._Histograms.keys()[0]][key2][phaseVarDict[key1]\
                  [key2]['i1']] = phaseVarDict[key1][key2]['Val']
            else:
               for key3 in phaseVarDict[key1][key2]:
                  self._Phases[self._Phases.keys()[0]][key1][key2][key3] = \
                     phaseVarDict[key1][key2][key3]


hapVary,hapDict,controlDict = G2stIO.GetHistogramPhaseData(self._Phases, \
                                 self._Histograms,Print=False)
histVary,histDict,controlDict = G2stIO.GetHistogramData(self._Histograms,Print=False)

if GSASIIpath.GetVersionNumber() > 1500:
   Natoms,atomIndx,phaseVary,phaseDict,pawleyLookup,FFtables,BLtables,maxSSwave = \
      G2stIO.GetPhaseData(self._Phases,self._restraintDict,self._rbIds,Print=False)
else:
   Natoms,atomIndx,phaseVary,phaseDict,pawleyLookup,FFtables,BLtables = \
      G2stIO.GetPhaseData(self._Phases,self._restraintDict,self._rbIds,Print=False)

self._parmDict.update(hapDict)
self._parmDict.update(histDict)
self._parmDict.update(phaseDict)

if parmVarDict:
   for name in parmVarDict:
      self._parmDict[name] = parmVarDict[name]

if controlVarDict:
   for name in controlVarDict:
      self._calcControls[name] = controlVarDict[name]
```

```
        '''
           #Load the parameters
           Histograms = self._Histograms
           varyList = self._varyList
           parmDict = self._parmDict
           Phases = self._Phases
           calcControls = self._calcControls
           pawleyLookup = self._pawleyLookup
           restraintDict = self._restraintDict
           rbIds = self._rbIds
        '''


#print 'Successful'
```
